# Surgical management of acute quadriceps tendon rupture (a case report with literature review)

**DOI:** 10.11604/pamj.2015.22.243.7533

**Published:** 2015-11-13

**Authors:** Badr Ennaciri, Eric Montbarbon, Emmanuel Beaudouin

**Affiliations:** 1 Department of Orthopedics, Avicenna University Hospital, Rabat, Morocco; 2 Department of Orthopedics, Chambéry Hospital, Chambéry, France

**Keywords:** Quadriceps tendon, tear, Krackow sutures

## Abstract

Quadriceps tendon rupture is uncommon and often overlooked in emergency. Tearing affects weakening tendon by systemic diseases or some medications. The mechanism is generally indirect. Inability to actively extend the knee associated to a supra-patellar defect evoke easily the diagnosis without other investigations. Surgical repair is realized in emergency to completely restore the extension. We report a case of a patient who has sustained of complete quadriceps tendon tear after a long period of tendon weakening by statin therapy, hypertension and diabetes. The repair has consisted on end-to-end Krackow sutures associated with bone suture to the proximal pole of the patella. Surgeons and emergency physicians must think to this form of extensor apparatus rupture, because early diagnosis leads to early treatment and to best outcomes.

## Introduction

Rupture of the extensor apparatus of the knee in adults is dominated by patellar fracture [1]. Quadriceps tendon ruptures are uncommon injuries, mainly affect patients over 40 years of age [2], in a context of systemic disease. Diagnosis is easily suggested by inability to actively extend the knee, but is still often overlooked in emergency. Usually, early surgical management is needed to reinsert the tendon at the superior aspect of the patella. We report a case of a patient who has sustained of an acute quadriceps tendon tear. The aim of this case, is to motivate emergency physicians and orthopedists to always evoke this diagnosis in emergency, especially, in aged patients with comorbidities.

## Patient and observation

**Figure 1:**
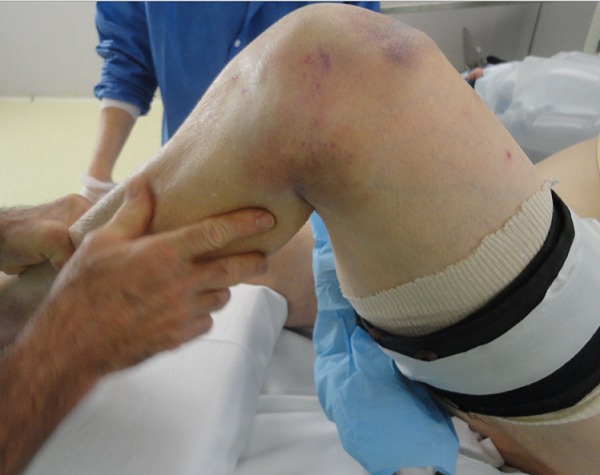
Skin ecchymosis in supra-patellar of the left knee

**Figure 2:**
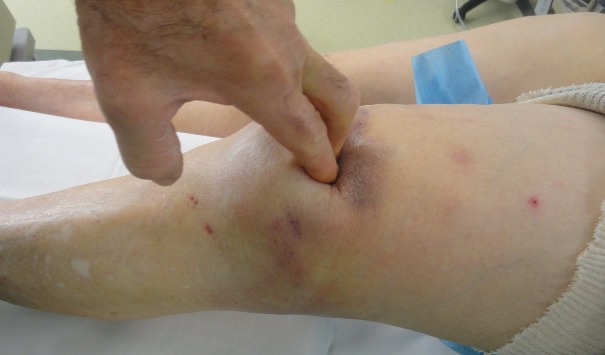
Knee examination showing depression in supra-patellar

**Figure 3:**
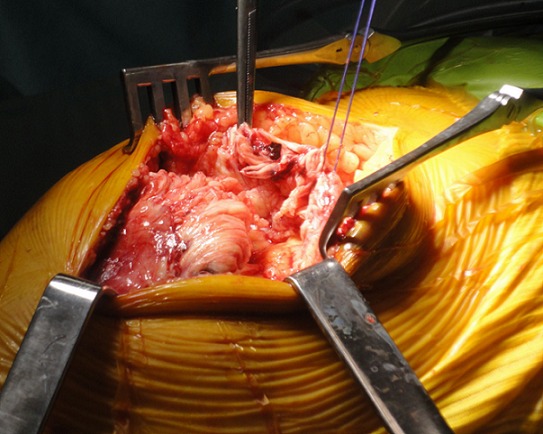
Surgical exploration showing complete rupture of quadriceps tendon onto the *vastus intermedius*

**Figure 4:**
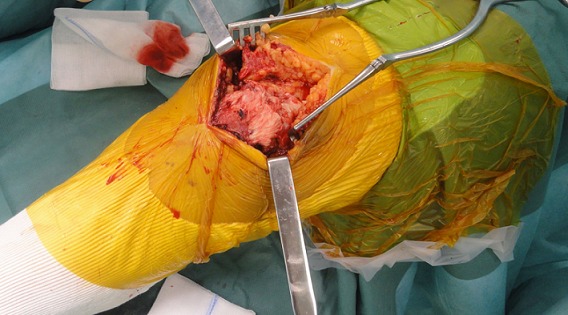
Krackow sutures associated with bone reinforcement of the tendon

A 83 years old women, hypertensive, diabetic and had dyslipidemia, cognitive disorders and total hip arthroplasty 10 years ago. She fell down the stairs at home and had sustained of a direct closed traumatism of her left knee. The patient suffered suddenly from a severe pain, total disability of the left limb and cracking sensation. The clinical exam showed skin ecchymosis (Figure 1) and palpable supra-patellar depression (Figure 2), active extension of the knee was impossible and neuro-vascular status of the left lower limb was normal. Radiograph of the knee showed a *patella baja* without patellar avulsion. Acute quadriceps tendon rupture was easily evoked. The patient was transferred to the operating room; after a vertical anterior approach centered on the patella, the tendon tear was confirmed (Figure 3) and an end-to-end Krackow sutures, associated with bone suture to the proximal pole of the patella were possible using Vicryl n° 2 (Figure 4).The knee was immobilized in a plaster to protect tendon repair for 6 weeks. Postoperative rehabilitation has consisted on a passive flexion and extension limited to 60° during 6 weeks followed by active motion after this period until total amplitude restoration.

## Discussion

Quadriceps tears represent the second injury to the extensor mechanism of the knee after patellar fractures [2], but still uncommon with an incidence of 1.37/100,000 per year [3]. Quadriceps lesions classically involve onto the *vastus intermedius aponeurosis* at the distal insertion. In elderly patients, tearing result usually from indirect traumatism by sudden quadriceps contraction, after a long time of tendon weakening due to previous injury or systemic disease (renal insufficiency, diabetes, rheumatoid polyarthritis, gout, hyperparathyroidism, disseminated erythematous lupus, or obesity) [1]. Tendon rupture associated with statin therapy is rare [4], operative view in our case, showed degenerative tendon. Pain, cracking sensation, active knee extension deficiency and palpable supra-patellar defect are the typical signs and found in about 60% of patients [5]. However, the diagnosis may be missed in emergency because of partial tear. Anterior-posterior and lateral radiograph of the knee can objectify supra-patellar soft tissue defect, joint effusion, *patella baja* or avulsion fragments [6]. Ultrasonography is used in the investigation of quadriceps tendon ruptures; it's a safe, non-invasive exam and allows dynamic assessment of the tendon [7], in our case, the diagnosis was evident clinically, without need to another investigation. Many repair techniques have been developed from simple suture with catgut to wire-reinforced repair, pull-out suture fixation through patella, suture anchor fixation, tendon lengthening repair, allograft, autograft and synthetic materials. Intra-tendon tear is relatively rare and is managed by end-to-end suture. Krackow suture was used in our case to obtain strong knots [8].

## Conclusion

The diagnosis of quadriceps tendon rupture must be considered in emergency if patient has a traumatism of the knee associated to active extension deficiency. Imaging should not substitutes a good clinical examination and surgical repair become too much easier in this situation.

## Competing interests

The authors declare no competing interests.

## Authors’ contributions

Badr Ennaciri: the corresponding author, contributed to patient's treatment and his clinical follow-up, conception and design, acquisition, analysis and interpretation of data, drafting the article, critical revision of the article, final approval of the version to be published. Eric Montbarbon: contributed to patient's treatment and his clinical follow-up, analysis and interpretation of data, drafting the article, critical revision of the article, final approval of the version to be published. Emmanuel Beaudouin: contributed to patient's treatment and his clinical follow-up, conception and design, acquisition, analysis and interpretation of data, drafting the article, critical revision of the article, final approval of the version to be published. All authors read and approved the final manuscript.
